# Cost-effectiveness study of early versus late parenteral nutrition in critically ill children (PEPaNIC): preplanned secondary analysis of a multicentre randomised controlled trial

**DOI:** 10.1186/s13054-017-1936-2

**Published:** 2018-01-15

**Authors:** Esther van Puffelen, Suzanne Polinder, Ilse Vanhorebeek, Pieter Jozef Wouters, Niek Bossche, Guido Peers, Sören Verstraete, Koen Felix Maria Joosten, Greet Van den Berghe, Sascha Cornelis Antonius Theodorus Verbruggen, Dieter Mesotten

**Affiliations:** 1 Intensive Care Unit, Department of Paediatrics and Paediatric Surgery, Erasmus Medical Centre, Sophia Children’s Hospital, Rotterdam, The Netherlands; 2 Department of Public Health, Erasmus Medical Centre, Rotterdam, The Netherlands; 3 Clinical Division and Laboratory of Intensive Care Medicine, Department of Cellular and Molecular Medicine, University Hospitals Leuven, Leuven, Belgium; 4 Department of Control and Compliance, Erasmus Medical Centre, Rotterdam, The Netherlands; 5 Department Medical Administration, University Hospitals Leuven, Leuven, Belgium

**Keywords:** Parenteral nutrition, Cost-effectiveness, Health economics, Costs, Intensive care

## Abstract

**Background:**

The multicentre randomised controlled PEPaNIC trial showed that withholding parenteral nutrition (PN) during the first week of critical illness in children was clinically superior to providing early PN. This study describes the cost-effectiveness of this new nutritional strategy.

**Methods:**

Direct medical costs were calculated with use of a micro-costing approach. We compared the costs of late versus early initiation of PN (*n* = 673 versus *n* = 670 patients) in the Belgian and Dutch study populations from a hospital perspective, using Student’s *t* test with bootstrapping. Main cost drivers were identified and the impact of new infections on the total costs was assessed.

**Results:**

Mean direct medical costs for patients receiving late PN (€26.680, IQR €10.090–28.830 per patient) were 21% lower (-€7.180, *p* = 0.007) than for patients receiving early PN (€33.860, IQR €11.080–34.720). Since late PN was more effective and less costly, this strategy was superior to early PN. The lower costs for PN only contributed 2.1% to the total cost reduction. The main cost driver was intensive care hospitalisation costs (-€4.120, *p* = 0.003). The patients who acquired a new infection (14%) were responsible for 41% of the total costs. Sensitivity analyses confirmed consistency across both healthcare systems.

**Conclusions:**

Late initiation of PN decreased the direct medical costs for hospitalisation in critically ill children, beyond the expected lower costs for withholding PN. Avoiding new infections by late initiation of PN yielded a large cost reduction. Hence, late initiation of PN was superior to early initiation of PN largely via its effect on new infections.

**Trial registration:**

ClinicalTrials.gov, NCT01536275. Registered on 16 February 2012.

**Electronic supplementary material:**

The online version of this article (doi:10.1186/s13054-017-1936-2) contains supplementary material, which is available to authorized users.

## Background

Healthcare costs are growing worldwide. Intensive care is responsible for a substantial proportion of all healthcare expenses, particularly prolonged intensive care and palliative care [1–4]. Intensive care costs are largely dependent on length of stay (LOS) in the intensive care unit (ICU), which is strongly influenced by complications, such as hospital-acquired infections [5].

Recently, a multicentre, randomised, controlled, parallel-group, superiority trial, with the acronym PEPaNIC (*n* = 1.440) concluded that withholding parenteral nutrition (PN) during the first week of critical illness in children was clinically superior to providing PN within 24 hours when enteral nutrition was insufficient [6], resulting in fewer patients with new infections. Aside from this clinical benefit, an additional economic benefit of late PN would be an extra argument for implementation of this new nutritional strategy.

Currently, no studies have investigated costs of different timing of initiation of PN in children in the paediatric ICU (PICU). Our cost-effectiveness analysis was predesigned, offering a unique opportunity for a micro-costing approach [7]. With this method of calculating hospital costs, all relevant cost categories are included and costs are calculated at the most detailed level per patient, in contrast to the gross-costing approach, whereby the cost categories are highly aggregated or only hospitalisation costs are included.

We hypothesised that withholding PN for one week is a cost-saving strategy comprising more than merely omitting the costs of PN itself. The aims of this study were (1) to compare total direct medical costs of early versus late initiation of PN in the PICU from a hospital perspective in an international context, (2) to provide detailed insight into the distribution of cost components, and (3) to assess the impact of acquiring a new infection in the PICU on direct medical costs.

## Methods

### Context

A total of 1440 critically ill children, aged 0 (term neonates) to 17 years, from three large tertiary referral PICUs in three countries (University Hospitals Leuven in Belgium, Erasmus MC in The Netherlands, and Stollery Children’s Hospital in Canada) were randomly assigned to early initiation of PN (standard care) or late initiation of PN (intervention). Initiation and dose of enteral nutrition (EN) and the administration of trace elements, minerals and vitamins were identical in both groups. Patients assigned to the group with late initiation of PN (*n* = 717) received no PN during the first week of critical illness. Patients assigned to the group with early initiation of PN (*n* = 723) received PN within 24 hours, according to the local standards. After the first week, when patients were still in the PICU and EN was insufficient to meet nutritional goals, PN was administered equally in both groups according to standard nutrition protocols [6, 8]. The institutional ethical review boards of the participating centres in Leuven (ML8052), Rotterdam (NL38772.000.12) and Edmonton (Pro00038098) approved the study, which was performed in accordance with the 1964 Declaration of Helsinki and its amendments. Written informed consent was obtained from the parents or legal guardians.

In this study, we explored the total direct medical costs, from a hospital perspective, in the Belgian and Dutch study populations, as these healthcare systems are reasonably comparable. Including the patients from Canada would introduce a bias, as cost calculations and reimbursements are too differently structured in Anglo-Saxon healthcare systems. Therefore, we excluded this centre from the cost analyses.

### Healthcare systems

In the Dutch healthcare system, hospitals are mainly paid by private insurance companies according to tariffs based on “Diagnosis Therapy Combination” (DBC) [9]. However, registered DBCs per patient do not represent individualised healthcare consumption. As the tariffs are fixed, specific healthcare activities are not presented in the patients’ invoices. Therefore, we used individual healthcare consumption and corresponding unit prices, which are registered by the hospital for reporting and stock management.

In Belgium, healthcare costs are reimbursed by sickness funds and private insurance companies. Since all healthcare activities are represented in the patients’ invoices, these invoices can be used to accurately quantify healthcare consumption. However, total healthcare costs are mainly covered by advance payments to the hospital, directly by the government. Consequently, for healthcare activities for which the hospital receives these advance payments, only 25% of the costs are represented in the patients’ invoices. When this is corrected to 100%, they reflect real healthcare costs from a hospital perspective [10].

### Resource consumption

The participating clinicians filled out standardised case report forms during the ICU stay, including duration of ICU dependency, post-ICU hospitalisation, mechanical ventilation, renal replacement therapy (RRT) and mechanical hemodynamic support. LOS encompassed both index and transferral hospitals. PN consumption was obtained from the study database for Dutch patients and from the invoices for Belgian patients. Detailed information on diagnostic procedures, medication, blood products, surgery and consultations were obtained from the data management system of the hospital for Dutch patients and from the invoices for Belgian patients.

Healthcare consumption was divided into ten cost categories: (1) ICU hospitalisation (both index and transferral hospital); (2) post-ICU hospitalisation (both index and transferral hospital); (3) PN; (4) medication; (5) laboratory diagnostics; (6) other diagnostics; (7) ventilator support; (8) RRT and mechanical haemodynamic support; (9) surgery; and (10) consultations from other specialists.

### Economic evaluation

The cost-effectiveness analyses were based on the Dutch and Belgian guidelines for performing costs studies [10, 11]. Furthermore, this study is in line with the international Consolidated Health Economic Evaluation Reporting Standards (CHEERS) statement [12]. Real medical costs were calculated by multiplying the volumes of healthcare use with the corresponding unit prices. Costs were calculated during two periods. From randomisation until ready-for-discharge from ICU, or death, the costs in all aforementioned cost categories were calculated. Ready-for-discharge was a priori chosen to avoid bias due to availability of beds on regular wards and was defined as “no longer requiring or no longer at risk for requiring vital organ support”. From ready-for-discharge from ICU until discharge from hospital, only hospitalisation costs were calculated. If a patient was transferred to another hospital, only hospitalisation costs were included for the period from discharge from the index hospital until discharge from the transferral hospital or death. Since the time horizon was less than one year, unit prices were not discounted.

In the Netherlands, the unit prices were available from the hospital’s financial database, and were adjusted to the year 2014. For hospital days (non-ICU), a national guiding price per day was used, because children were referred to different hospitals, charging different prices [11]. The daily costs of mechanical ventilation and RRT were estimated based on published literature [13, 14]. Production costs of infusions for the intervention group were calculated by summing the costs of the PN ingredients, pharmacy compounding costs and additional trial intervention costs.

In Belgium, financial data were registered by the billing and warehousing collaborators of the index hospital as this is standard procedure for invoicing. The unit prices were official, nationally fixed prices adjusted to the year 2014, and were converted to 100%, if necessary, to obtain real costs from a hospital perspective [10]. There were no additional trial intervention costs for infusions in the group receiving late initiation of PN.

Costs of medication were categorised according to the first level of the Anatomical Therapeutic Chemical (ATC) classification, which is the World Health Organisation (WHO) tool for drug utilisation research [15]. Each drug has its unique ATC code and price. Costs for ATC code B05BA (PN solutions) were reported separately. Since we were unable to distinguish costs per ATC code in Dutch patients, we excluded them from this ATC code analysis. However, since new infections were a primary outcome in the trial, we analysed the costs of anti-infective drugs in both centres.

### Study endpoints

The primary endpoint was the difference in total direct medical costs, from a hospital perspective, between early and late initiation of PN. Furthermore, the ten cost categories were analysed separately. In order to give insight into costs among different groups of patients, we compared total direct medical costs of early initiation of PN with late initiation of PN in the stratification groups as used for the PEPaNIC trial: “Surgical cardiac”, “Surgical other”, “Medical neurological” and “Medical other”, and age groups younger and older than one year [8]. Additionally, the drugs responsible for differences in medication costs were investigated based on the ATC codes. Also, the impact of new infections on total costs was calculated. Finally, we explored the cost-effectiveness of late initiation of PN, using the number of patients with a new infection prevented in the ICU as an effect measure.

### Statistical analyses

The PEPaNIC trial was a priori statistically powered to detect a difference in new infections. Therefore, the statistical power to detect differences in total direct medical costs was dependent on the number of patients enrolled in the original PEPaNIC trial. This cost analysis was an a priori planned secondary analysis.

Costs were reported in euro (€), as mean (SD and IQR), as recommended for cost analyses [16]. IQR was reported, as cost data is always highly skewed, and IQR reflects the statistical dispersion more realistically than standard deviation or standard error. Other data were reported as mean (SE), median (IQR) or number (%), as appropriate. In order to check whether the major costs were similarly distributed into the cost categories in both centres, a Pareto analysis was performed. This is a chart to demonstrate which factors are contributing most to a problem (i.e. total costs) [17].

Costs were compared univariably using Student’s one-tailed *t* test with bootstrapping (×1000) [16], LOS was compared using the Mann-Whitney U test, and the incidence of new infection was compared using Fisher’s exact test. Based on the clinical results that point out clearly that late PN reduces resource consumption by reduction of new infections and shorter PICU stay, we have chosen to test the differences in costs one-sided, hypothesising that late PN is less costly than early PN. One-sided *p* values <0.05 were considered statistically significant. Effects were reported as mean difference or odds ratio (OR) with the corresponding 95% confidence interval (CI). The OR for acquiring a new infection was adjusted for age, risk of malnutrition (STRONGkids group), treatment centre, admission diagnosis, and degree of organ failure (PeLOD score), in line with the PEPaNIC trial [6], and also Paediatric Index of Mortality 2 (PIM2) score to adjust for risk of mortality. The adjusted OR was analysed using binary logistic regression. Analyses were conducted using IBM SPSS statistics, version 24.0.

### Sensitivity analyses

Sensitivity analyses were conducted as follows:

1. The total costs were analysed using prices from the Belgian healthcare system for all patients.
2. The total costs were analysed using prices from the Dutch healthcare system for all patients.
3. The total costs were analysed separately in the Belgian patients.
4. The total costs were analysed separately in the Dutch patients.
5. As only the hospitalisation costs of the post-ICU period were included in the primary analysis, the additional post-ICU costs (i.e. laboratory, medication costs) were left out. Since this could underestimate our results, the estimated additional post-ICU costs were added in the third sensitivity analysis. These additional post-ICU costs were estimated based on the invoices of the Belgian patients.

## Results

We compared the total direct medical costs of late initiation of PN (N = 673 patients) with those of early initiation of PN (N = 670 patients) in the Dutch and Belgian study populations. The patients’ baseline characteristics and main clinical outcomes are described in Table 1.

**Table 1:** Baseline characteristics and main clinical outcomes

	Early PN (*N* = 670)	Late PN (*N* = 673)	
Baseline characteristics^a^			
Median age (IQR), years	1.3 (0.3–6.0)	1.4 (0.2–7.0)	
Age <1 year, *n* (%)	311 (46.4)	312 (46.4)	
Male sex, *n* (%)	386 (57.6)	393 (58.4)	
STRONGkids risk level, *n* (%)^b^			
Medium	593 (88.5)	600 (89.2)	
High	77 (11.5)	73 (10.8)	
Median PeLOD score, first 24 hours in paediatric ICU (IQR)^c^	21 (12–32)	21 (11–31)	
Median PIM2 score (IQR)^d^	-2.8 (-3.7; -1.3)	-2.8 (-3.7; -1.6)	
Emergency admission, *n* (%)	325 (48.5)	308 (45.7)	
Diagnostic group, *n* (%)			
Surgical cardiac	264 (39.4)	259 (38.5)	
Surgical other	202 (30.1)	205 (30.4)	
Medical neurological	44 (6.6)	50 (7.4)	
Medical other	160 (23.9)	159 (23.5)	
Condition on admission, *n* (%)			
Mechanical ventilation required	596 (90.0)	587 (87.2)	
ECMO or other mechanical hemodynamic support required	16 (2.4)	22 (3.3)	
Infection	256 (38.2)	244 (36.3)	
Clinical primary outcomes			*P* value^e^
New infections, *n* (%)	120 (17.9)	71 (10.6)	<0.001
Median duration of stay in paediatric ICU (IQR), days^e^	4 (2–9)	3 (2–7)	0.002

*PN* parenteral nutrition, *STRONGkids* Screening Tool for Risk on Nutritional Status and Growth, *PeLOD* Paediatric Logistic Organ Dysfunction, *PIM2* Paediatric Index of Mortality 2, *ECMO* extracorporeal membrane oxygenation, *ICU* intensive care unit

^a^There were no significant differences in characteristics between treatment groups at baseline

^b^STRONGkids scores range from 0 to 5, with a score of 0 indicating a low risk of malnutrition, a score of 1 to 3 indicating medium risk, and a score of 4 to 5 indicating high risk

^c^PeLOD scores range from 0 to 71, with higher scores indicating more severe illness

^d^PIM2 scores, with higher scores indicating a higher risk of mortality

^e^The duration of stay in the paediatric ICU was defined as the time from admission until the patient was ready for discharge (i.e., the patient no longer required or was no longer at risk of requiring vital-organ support). The duration of stay was not censored, nor adjusted for death

### Total healthcare costs and evaluation of cost drivers

Late initiation of PN, as compared with early initiation of PN, reduced the mean total direct medical costs by €7.180 (95% CI (-€12.920; -€1.880), *p* = 0.007) per patient (early initiation of PN €33.860, late initiation of PN €26.680), which is a saving of 21% (Table 2).

**Table 2:** Healthcare costs split by major cost categories

Cost category	Early PN, €			Late PN, €			Mean difference (95% CI), €	*P* value (*t* test)
	Mean	SD	p25–p75	Mean	SD	p25–p75		
ICU hospitalisation	13.710	35.130	2.270–13.250	9.590	16.730	2.270–9.070	-4.120 (-7.590; -1.500)	0.003
Medication	1.810	7.430	250–1000	1.160	3.080	220–830	-650 (-1.360; 100)	0.03
Ventilator support	1.740	6.650	150–1.430	1.100	2.300	150–1.070	-640 (-1.260; -190)	0.03
Post-ICU hospitalisation	7.140	14.530	1.500–6.900	6.560	14.270	1.500–6.270	-580 (-2.100; 1.080)	0.24
Surgery	4.760	5.480	280–6.500	4.240	4.260	200–6.280	-520 (-1.080; 10)	0.03
Laboratory diagnostics	2.290	4.090	540–2.160	1.820	3.370	440–1.820	-460 (-860; -40)	0.01
PN	300	500	60–300	150	270	20–170	-150 (-200; -110)	<0.001
Other diagnostics	690	2.020	80–550	610	1.610	80–430	-80 (-290; -110)	0.20
Consultations	470	690	40–680	440	630	30–680	-30 (-100; 50)	0.19
RRT and mechanical hemodynamic support	950	6.740	0–0	1.010	9.370	0–0	60 (-810; 1.020)	0.45
Total	33.860	57.610	11.080–34.720	26.680	35.850	10.090–28.830	-7.180 (-12.920; -1.880)	0.007

Cost categories were ranked according to the mean difference between the treatment groups

*PN* parenteral nutrition, *CI* confidence interval, *ICU* Intensive Care Unit, *RRT* renal replacement therapy, *p25–p75* 25th to 75th percentile

The major costs were divided into cost categories similarly for both centres (Additional file 1). Differences in mean costs between Belgian and Dutch patients were due to shorter duration of stay in the ICU (factor 0.55) in Belgian patients (*p* < 0.001), which might be caused by differences in patient populations. In contrast, the Belgian costs per day in ICU (mean costs of all categories summed, except hospitalisation costs post-ICU, divided by the duration of ICU stay) were higher (factor 1.18) than the Dutch costs (*p* < 0.001). Almost all cost categories showed lower costs with late initiation of PN than with early initiation of PN. The largest reduction was in ICU hospitalisation costs (-€4.120, 95% CI (-€7.590; -€1.500)), medication costs (-€650, 95% CI (-€1.360; €100)), and ventilator support costs (-€640, 95% CI (-€1.260; -€190)) (Table 2). This reduction in costs is in line with the shorter ICU stay in the group receiving late initiation of PN (Table 1). PN costs were responsible for 2.1% (-€150, 95% CI (-€200; -€110)) of the reduction in total costs.

The age category (<1 year versus ≥1 year) was not apparently related to proportional cost reduction with late initiation of PN (Additional file 2). Patients admitted for a medical reason other than neurological disease had the largest cost reduction with late initiation of PN (-€14.720, 95% CI (-€30.720; €130), *p* = 0.04) (Additional file 3). Patients admitted for non-cardiac surgery also had cost reduction with late initiation of PN (-€9.490, 95% CI (-€20.720; €1.040), *p* = 0.05) (Additional file 3). Furthermore, 26% of the patients had a prolonged ICU stay (>7 days), accounting for 60% of the costs. Moreover, the most expensive 1% of patients accounted for 13% of the total costs.

The distribution of medication costs is described in Table 3. The combination of medications of category B (blood and blood forming organs), containing PN solutions, and category J (anti-infectives), containing antibiotics, was responsible for 80% of the reduction in medication costs with late initiation of PN (Table 3).

**Table 3:** Medication costs of Belgian patients split by Anatomical Therapeutic Classification (ATC) system classes

ATC code	Early PN, €			Late PN, €			Mean difference (95% CI), €	*P* value (*t* test)
	Mean	SD	p25–p75	Mean	SD	p25–p75		
B (blood/blood forming organs)	850	1.820	210–740	580	1.350	140–480	-270 (-510; -40)	0.02
J (anti-infectives)^a^	320	2500	4–90	170	1.030	4–50	-150 (-470; 80)	0.17
A (alimentary tract/metabolism)	70	250	20–70	50	80	10–40	-30 (-40; -10)	<0.001
V (various)	100	140	20–140	80	110	20–110	-20 (-40; -7)	0.004
N (nervous system)	90	180	30–80	70	150	30–80	-20 (-40; 0)	0.03
C (cardiovascular)	55	150	3–50	45	100	1–50	-10 (-30; 5)	0.07
R (respiratory)	20	150	0–0	10	50	0–0	-10 (-30; 7)	0.10
H (hormonal)	30	110	0–30	20	60	0–30	-10 (-20; 3)	0.10
M (musculo-skeletal)	13	40	3–10	7	10	3–9	-5 (-10; -2)	0.01
D (dermatologics)	10	70	0–8	7	30	0–8	-3 (-10; 3)	0.26
S (sensory organs)	3	5	0–4	2	3	0–4	-1 (-1; 0)	0.01
P (antiparasitic)	1	20	0–0	0	0	0–0	1 (-3; 0)	0.19
G (genito-urinary/sex hormones)	1	8	0–0	1	8	0–0	0 (-1; 1)	0.48
L (antineoplasmic/immunomodulating)	25	330	0–0	45	320	0–0	20 (-30; 60)	0.26
Total	1.600	4.663	380–1.260	1.070	2.577	310–830	-500 (-1.060; 20)	0.03

Cost categories were ranked according to the mean difference between the treatment groups. *PN* parenteral nutrition, *CI* confidence interval, *p25–p75* 25th to 75th percentile

^a^Data from both Belgian and Dutch patients

### Impact of new infections

**Fig 1:**
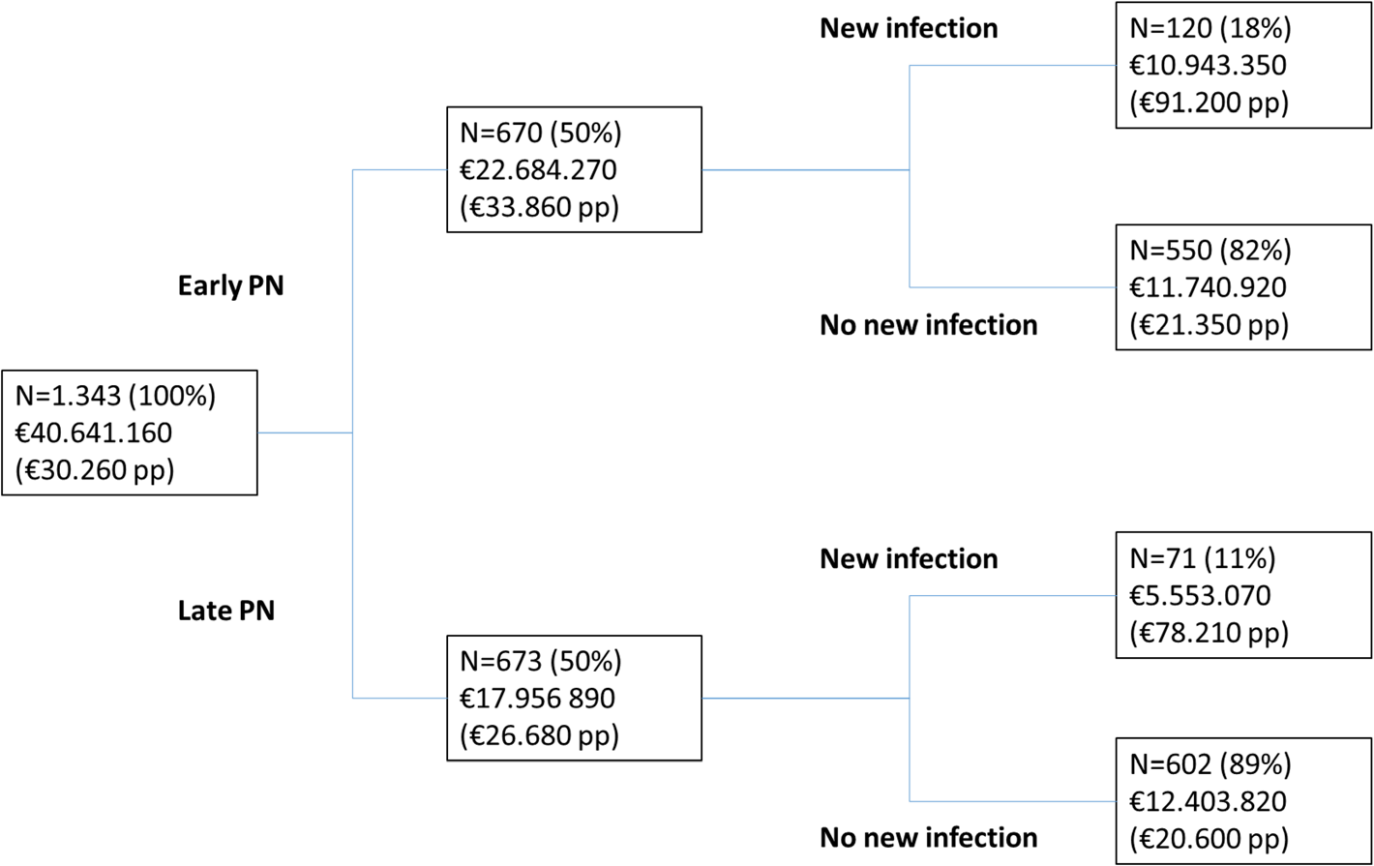
Costs of patients with or without new infections. *PN* parenteral nutrition, *pp* per patient

The proportion of patients with a new infection acquired in the ICU was smaller with late initiation of PN than with early initiation of PN (patients included for cost analysis, 10.6% and 17.9%, respectively, *p* < 0.001) (Table 1), with a corresponding adjusted odds ratio of 0.51 (95% CI (0.36; 0.71)). In the whole group, 1.2% of the least expensive 50% of patients had acquired a new infection compared to 28.5% of the most expensive 50% of patients. Moreover, 85% (11 patients) of the most expensive 1% of patients (13 patients) had acquired a new infection. Figure 1 depicts the cost tree of patients with and without a new infection in both randomisation groups. In patients who acquired a new infection, costs were increased from €21.350 to €91.200 (difference €69.850, 95% CI (€50.700; €91.560), *p* = 0.001) with early initiation of PN and from €20.600 to €78.210 (difference €57.610, 95% CI (€41.890; €73.970), *p* = 0.001) with late initiation of PN, predominantly caused by ICU hospitalisation costs (early-PN group, difference €37.210, 95% CI (€26.200; €52.750), *p* = 0.002; late-PN group: difference €27.530, 95% CI (€20.660; €34.940), *p* = 0.001).

Late initiation of PN was more effective and less costly than early initiation of PN, and falls into the south-eastern quadrant of the cost-effectiveness plane. Interventions in this quadrant are always considered cost-effective [18, 19].

### Sensitivity analyses

The primary results were robust, as they could be reproduced with multiple sensitivity analyses (Table 4). Using the Dutch or Belgian unit prices for all patients showed a cost difference of late versus early PN of -€8.690 respectively -€6.090, which is within the 95% CI of the primary analysis of the base case ((-€12.920; -€1.880). Also, when analysing the Dutch and Belgian patients separately, the cost reduction with late PN was within the 95% CI of the primary analysis of the base case (Table 4). The difference in absolute costs between the centres was predominantly due to more resource consumption in the Dutch patients (i.e. longer LOS) (Additional file 4). In the third sensitivity analysis, total post-ICU costs were estimated and added to the total ICU costs. Post-ICU costs were predominantly hospitalisation costs (85%), which were already included in the base case. Consequently, the additional post-ICU costs of approximately €1.100 in this sensitivity analysis represented the 15% of post-ICU costs that were not included in the base case.

**Table 4:** Sensitivity analyses

	Early PN, €				Late PN, €				Mean difference (95% CI), €	*P* value (*t* test)
	*N*	Mean	SD	p25–p75	*N*	Mean	SD	p25–p75		
Primary analysis (total ICU costs and post-ICU hospitalisation costs)	670	33.860	57.600	11.080–34.720	673	26.680	35.850	10.090–28.830	-7.180 (-12.920; -1.880)	0.007
Sensitivity analyses										
1. Belgian unit prices^a^	670	28.380	41.210	10.460–29.880	673	22.290	24.960	9.320–25.390	-6.090 (-9.950; -2.480)	<0.001
2. Dutch unit prices^b^	670	37.960	62.120	12.570–38.350	673	29.270	37.180	11.560–32.390	-8.690 (-14.090; -3.190)	0.003
3. Belgian patients	373	22.930	22.460	10.060–23.780	377	17.600	15.920	9.150–19.700	-5.330 (-8.650; -2.360)	0.003
4. Dutch patients	297	47.580	78.820	14.860–46.410	296	38.250	48.630	12.790–42.380	-9.330 (-20.410; 1.250)	0.04
5. Total ICU and total post-ICU costs^c^	670	34.990	58.316	11.470–35.580	673	27.800	36.859	10.530–30.580	-7.190 (-12.420; -1.970)	0.002

*PN* parenteral nutrition, *CI* confidence interval, *ICU* Intensive Care Unit

^a^The total costs were analysed using prices from the Belgian healthcare system for all patients

^b^The total costs were analysed using prices from the Dutch healthcare system for all patients

^c^In the primary analysis, only the hospitalisation costs of the post-ICU period were included. The estimated additional post-ICU costs (i.e. laboratory, medication) were added. These additional post-ICU costs were estimated based on the invoices of the Belgian patients

## Discussion

This cost-effectiveness study of the PEPaNIC trial showed that the total direct medical costs were considerably lower when PN was withheld during the first week of critical illness in children as compared with early initiation of PN. This cost reduction was mainly due to lowering of ICU hospitalisation costs, although most cost components were reduced by not using early PN. The reduction of the costs for PN was responsible for only 2.1% of the total cutback of costs, which supported our hypothesis that the health-economic impact of withholding PN encompassed more than the omission of costs for PN itself. Taking into account the beneficial clinical impact of late initiation of PN, we can conclude that withholding PN in the first week of critical illness is superior to early initiation of PN largely by preventing new infections, which is cost-saving [18, 19].

Our results confirmed previously published results of studies in critically ill adults that have compared early with late initiation of PN [20, 21]. The American Thoracic Society has included “‘withholding PN for one week in critically ill adults” in the top five recommendations to improve healthcare while reducing healthcare costs [22]. One other cost analysis of the timing of PN in adults identified no difference in LOS, and US$ 3.170 higher costs per patient with late initiation of PN. However, the estimated costs in this study were based on a Monte Carlo simulation, in which the estimated probabilities of events, such as mechanical ventilation, have a large impact on cost differences [23]. A micro-costing approach, used in our study, provides more precise and more reliable results, as this method uses the real costs that have been incurred.

Three studies of paediatric ICU costs have been previously published, which allow comparison with our study results. First, Harron et al. reported ICU stay and direct ICU costs that are comparable to those we reported here, which supports the generalisability of the findings of our study [24]. Second, the CHiP study reported hospital costs during a 12-month period (~ £21.000) [4] that were slightly lower than those found in our study. These differences could be explained by different study populations, with more patients included after cardiac surgery, which may incur lower costs than medical or non-cardiac surgical paediatric ICU patients, and more patients with less organ failure, reflected by mean PeLOD of 7.5 as compared with a median PeLOD of 21 in our study. Third, Morillo-García et al. reported higher costs for children with a nosocomial infection, as compared with those without a new infection [25], which supports our conclusion that healthcare costs can be reduced by preventing new infections.

In line with previous research [26], we observed that the duration of ICU stay had a major effect on the costs. This was also reflected in the finding that patients with a prolonged ICU stay, the minority of the total patient population that was included, accounted for the majority of the costs.

The reduction in medication costs with late versus early initiation of PN was mainly due to lower use of products in ATC categories B (blood and blood-forming organs) and J (anti-infectives). This corroborates the finding that late initiation of PN reduces the proportion of patients with new infections, as it also does in adults [21]. Additionally, patients with a new infection had higher total costs per patient than those without a new infection. The fact that the proportion of patients with new infections increased from 1.2% among the least expensive patients to 85% among the 1% most expensive patients pointed to an effect of new infections on the total costs. Therefore, reducing the number of ICU days by preventing the occurrence of new infections by late initiation of PN seems to have had most influence on the cost reduction.

The strength of this study is the micro-costing approach, reflecting real costs that incurred. Additionally, our findings appeared robust across two healthcare systems. We have carefully checked whether combining the Dutch and Belgian populations would compromise our results by performing sensitivity analyses, which have shown unanimously a cost reduction with late PN, which was well within the range of the confidence interval of the base case. These results may support that the international character of this study increased external validity and, possibly, applicability to other European countries.

Some limitations should also be addressed. First, the Dutch daily costs of mechanical ventilation and renal replacement therapy had to be estimated, based on published literature on ICU costs [13, 14]. Second, the time horizon was limited to the hospital period and direct medical costs, and thus, the economic consequences could not be fully captured. One Swiss study, investigating out-of-pocket expenses of families with a child spending > 4 days in the ICU reported mean 86 (±31) Franc (converted ~ €137) per day, mainly on travel costs and meals [27]. As such, the full impact of late initiation of PN from a societal perspective could not be assessed. Third, quality-adjusted life years (QALYs) were not used as an outcome measure, as the time horizon was too short (hospital stay) to meaningfully assess long-term quality of life of critically ill children. It is acknowledged that a cost-utility analysis is preferred over a cost-effectiveness analysis, if a treatment has an impact on health-related quality of life. At this time, a long-term follow-up study is ongoing, with patients being evaluated 2 and 4 years after randomisation. This long-term follow-up study includes an assessment of quality of life. However, the aim of the current study was to perform a cost-effectiveness analysis and not a cost-utility analysis, which requires quality of life data. Finally, the lack of detailed drug costs for Dutch patients may have biased, and possibly underestimated, the differences in drug costs.

## Conclusion

The cost analyses showed that late initiation of PN reduced the direct medical costs by 21% in critically ill children as compared with early initiation of PN, beyond the expected lower costs for the use of PN itself. Avoiding new infections by late initiation of PN yielded a large cost reduction. Withholding PN during the first week of critical illness in children can thus be recommended both from a clinical and a health-economic perspective.

## Additional files

Additional file 1: Pareto charts of the cost categories in Belgian and Dutch patients, shown separately. (DOC 183 kb)

Additional file 2: Table showing total healthcare costs split by age into two groups. (DOC 28 kb)

Additional file 3: Table showing total healthcare costs split by diagnosis into four groups. (DOC 31 kb)

Additional file 4: Table showing resource utilisation and costs per centre separately in Belgian and Dutch patients respectively. (DOC 37 kb)

## Abbreviations

€: Euro
ATC classification: Anatomical therapeutical chemical classification
CI: Confidence interval
DBC: Diagnosis Therapy Combination (translation from Dutch)
ECMO: Extracorporeal membrane oxygenation
ICU: Intensive care unit
IQR: Interquartile range
LOS: Length of stay
OR: Odds ratio
PeLOD: Paediatric Logistic Organ Dysfunction
PIM2: Paediatric Index of Mortality 2
PN: Parenteral nutrition
RRT: Renal replacement therapy
SE: Standard error
STRONGkids: Screening Tool for Risk on Nutritional Status and Growth
US$: American Dollar
WHO: World health organisation
