# Supplementary material for: Cost-effectiveness study of early versus late parenteral nutrition in critically ill children (PEPaNIC): preplanned secondary analysis of a multicentre randomised controlled trial
Source: Crit Care. 2018 Jan 15;22:4. doi: 10.1186/s13054-017-1936-2 (PMC5769527; doi:10.1186/s13054-017-1936-2)
Supplement: Supplementary file 1 — Pareto charts of the cost categories in Belgian and Dutch patients, shown separately. (DOC 183 kb) [file 13054_2017_1936_MOESM1_ESM.doc]

**Additional file 1. Pareto charts of the cost categories**

a. Belgian patients b. Dutch patients


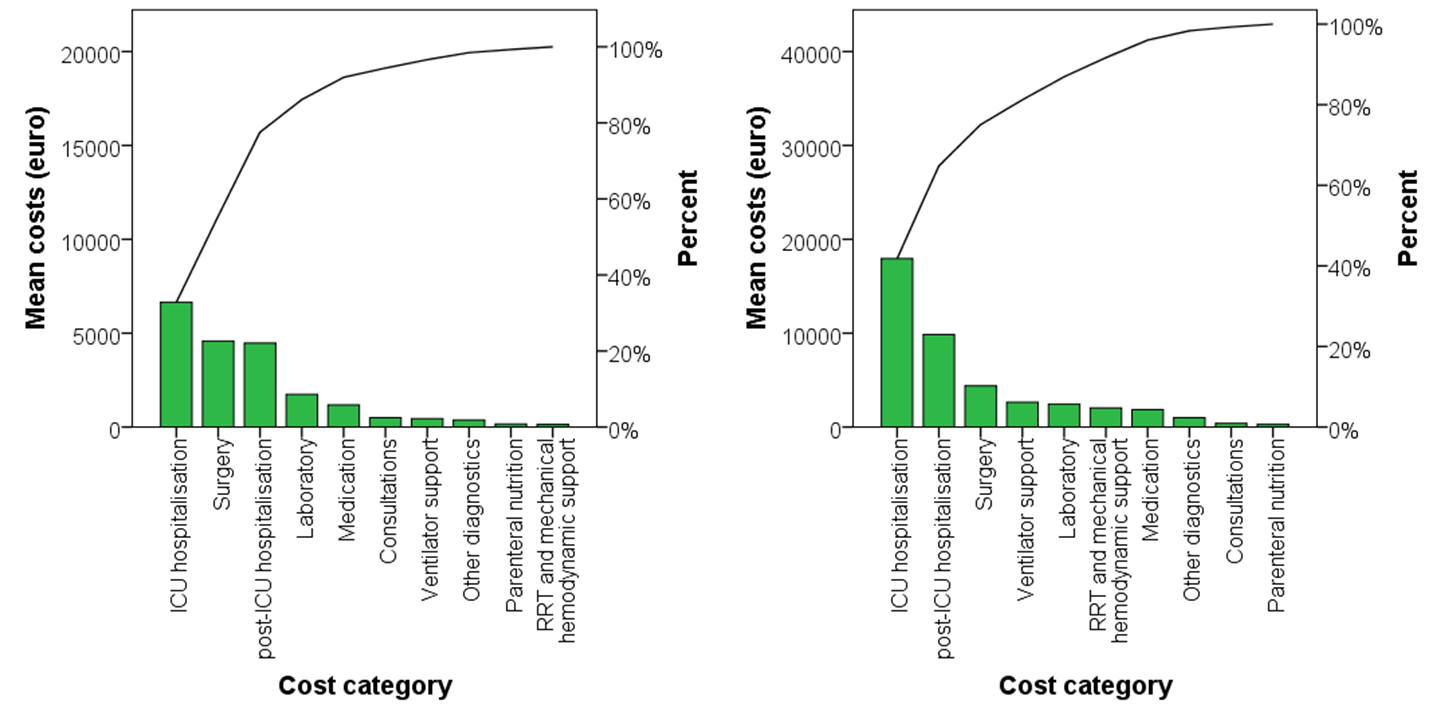


ICU = Intensive Care Unit, RRT = renal replacement therapy

The continuous lines reflect the cumulative total costs.
