# Supplementary material for: Cost-effectiveness study of early versus late parenteral nutrition in critically ill children (PEPaNIC): preplanned secondary analysis of a multicentre randomised controlled trial
Source: Crit Care. 2018 Jan 15;22:4. doi: 10.1186/s13054-017-1936-2 (PMC5769527; doi:10.1186/s13054-017-1936-2)
Supplement: Supplementary file 2 — Table showing total healthcare costs split by age into two groups. (DOC 28 kb) [file 13054_2017_1936_MOESM2_ESM.doc]

**Additional file 2. Total healthcare costs split by age**

| **Age** | **Early PN, €** | | | | **Late PN, €** | | | | **Mean difference [95%CI], €** | **P-value (*t* test)** |
| --- | --- | --- | --- | --- | --- | --- | --- | --- | --- | --- |
|  | N | Mean | SD | p25-p75 | N | Mean | SD | p25-p75 |  |  |
| Age 0-1 year | 311 | 39.240 | 69.030 | 12.580-38.980 | 312 | 30.700 | 38.790 | 12.000-31.130 | -8.540 [-18.520;110] | 0.04 |
| Age ≥1 year | 359 | 29.200 | 45.030 | 9.780-29.420 | 361 | 23.210 | 32.750 | 9.270-25.580 | -5.990 [-12.040;-330] | 0.02 |

Cost categories were ranked according to the mean difference between the treatment groups. CI = confidence interval, PN = parenteral nutrition
