# Supplementary material for: Cost-effectiveness study of early versus late parenteral nutrition in critically ill children (PEPaNIC): preplanned secondary analysis of a multicentre randomised controlled trial
Source: Crit Care. 2018 Jan 15;22:4. doi: 10.1186/s13054-017-1936-2 (PMC5769527; doi:10.1186/s13054-017-1936-2)
Supplement: Supplementary file 3 — Table showing total healthcare costs split by diagnosis into four groups. (DOC 31 kb) [file 13054_2017_1936_MOESM3_ESM.doc]

**Additional file 3. Total healthcare costs split by diagnosis group**

| **Diagnosis group** | **Early PN, €** | | | | **Late PN, €** | | | | **Mean difference [95%CI], €** | **P-value**  **(*t* test)** |
| --- | --- | --- | --- | --- | --- | --- | --- | --- | --- | --- |
|  | N | Mean | SD | p25-p75 | N | Mean | SD | p25-p75 |  |  |
| Medical - Other | 160 | 52.710 | 81.940 | 13.460-53.250 | 159 | 38.000 | 56.230 | 10.810-39.300 | -14.710 [-30.720;130] | 0.04 |
| Surgery - Other | 202 | 34.280 | 66.380 | 10.770-35.890 | 205 | 24.800 | 28.430 | 8.660-28.880 | -9.480 [-20.720;-1.040] | 0.05 |
| Surgery - Cardiac | 264 | 23.740 | 23.840 | 11.030-27.440 | 259 | 21.730 | 21.930 | 10.680-22.830 | -2.010 [-5.850;1.730] | 0.16 |
| Medical - Neurological | 44 | 24.030 | 26.000 | 7.470-25.800 | 50 | 24.090 | 31.090 | 7.630-29.520 | +60 [-10.630;11.160 | 0.50 |

Cost categories were ranked according to the mean difference between the treatment groups. CI = confidence interval, PN = parenteral nutrition
