# Supplementary material for: Cost-effectiveness study of early versus late parenteral nutrition in critically ill children (PEPaNIC): preplanned secondary analysis of a multicentre randomised controlled trial
Source: Crit Care. 2018 Jan 15;22:4. doi: 10.1186/s13054-017-1936-2 (PMC5769527; doi:10.1186/s13054-017-1936-2)
Supplement: Supplementary file 4 — Table showing resource utilisation and costs per centre separately in Belgian and Dutch patients respectively. (DOC 37 kb) [file 13054_2017_1936_MOESM4_ESM.doc]

**Additional file 4. Resource utilisation and costs per centre.**

| Centre | Leuven, Belgium  N=673 | Rotterdam, The Netherlands  N=670 |
| --- | --- | --- |
| ***Resource utilisation*** | | |
| Duration ICU (days) – mean (SE) | 5.9 (0.4) | 10.8 (1.0) |
| Duration post-ICU (days) – mean (SE) | 9.0 (0.7) | 15.7 (1.2) |
| Duration ventilator support (days) – mean (SE) | 3.7 (0.2) | 7.4 (0.8) |
| Duration renal replacement therapy (days) – mean (SE) | 0.2 (0.1) | 0.4 (0.1) |
| Duration mechanical hemodynamic support (days) – mean (SE) | 0.4 (0.1) | 0.7 (0.2) |
| ***Costs*** | | |
| ICU hospitalisation (euro) – mean (SD, IQR) | 6.650 (11.110, 1.980-6.800) | 17.950 (38.650, 3.310-18.220) |
| Post-ICU hospitalisation (euro) – mean (SD, IQR) | 4.470 (9.220, 1.500-3.990) | 9.860 (18.600, 1.880-11.600) |
| PN (euro) – mean (SD, IQR) | 160 (350, 20-150) | 300 (470, 100-300) |
| Medication (euro) – mean (SD, IQR) | 1.180 (3.540, 300-850) | 1.870 (7.560, 160-970) |
| Laboratory diagnostics (euro) – mean (SD, IQR) | 1.740 (2.460, 610-1.770) | 2.450 (4.900, 200-2.640) |
| Other diagnostics (euro) – mean (SD, IQR) | 380 (590, 110-380) | 990 (2.630, 0-760) |
| Ventilator support (euro) – mean (SD, IQR) | 450 (690, 150-440) | 2.640 (2.640, 710-2.490) |
| Renal replacement therapy and mechanical hemodynamic support (euro) – mean (SD, IQR) | 150 (1460, 0-0) | 2.050 (12.090, 0-0) |
| Surgery (euro) – mean (SD, IQR) | 4.580 (3940, 1.230-6.230) | 4.400 (5.920, 0-6.980) |
| Consultations (euro) – mean (SD, IQR) | 500 (360, 180-710) | 400 (900, 0-440) |
| Total (euro– mean (SD, IQR) | 20.250 (22.550, 9.570-21.160) | 42.920 (65.630, 13.580-43.380) |

ICU= intensive care unit, PN = parenteral nutrition
